# Expression of the Long Non-Coding RNA HOTAIR Correlates with Disease Progression in Bladder Cancer and Is Contained in Bladder Cancer Patient Urinary Exosomes

**DOI:** 10.1371/journal.pone.0147236

**Published:** 2016-01-22

**Authors:** Claudia Berrondo, Jonathan Flax, Victor Kucherov, Aisha Siebert, Thomas Osinski, Alex Rosenberg, Christopher Fucile, Samuel Richheimer, Carla J. Beckham

**Affiliations:** 1 University of Rochester Department of Urology, Strong Memorial Hospital Rochester, New York, United States of America; 2 University of Rochester School of Medicine and Dentistry, Rochester, New York, United States of America; 3 Division of Immunology and Rheumatology, University of Rochester, Strong Memorial Hospital Rochester, New York, United States of America; University of Navarra, SPAIN

## Abstract

Exosomes are 30-150nM membrane-bound secreted vesicles that are readily isolated from biological fluids such as urine (UEs). Exosomes contain proteins, micro RNA (miRNA), messenger RNA (mRNA), and long non-coding RNA (lncRNA) from their cells of origin. Although miRNA, protein and lncRNA have been isolated from serum as potential biomarkers for benign and malignant disease, it is unknown if lncRNAs in UEs from urothelial bladder cancer (UBC) patients can serve as biomarkers. lncRNAs are > 200 nucleotide long transcripts that do not encode protein and play critical roles in tumor biology. As the number of recognized tumor-associated lncRNAs continues to increase, there is a parallel need to include lncRNAs into biomarker discovery and therapeutic target algorithms. The lncRNA HOX transcript antisense RNA (HOTAIR) has been shown to facilitate tumor initiation and progression and is associated with poor prognosis in several cancers. The importance of HOTAIR in cancer biology has sparked interest in using HOTAIR as a biomarker and potential therapeutic target. Here we show HOTAIR and several tumor-associated lncRNAs are enriched in UEs from UBC patients with high-grade muscle-invasive disease (HGMI pT2-pT4). Knockdown of HOTAIR in UBC cell lines reduces *in vitro* migration and invasion. Importantly, loss of HOTAIR expression in UBC cell lines alters expression of epithelial-to-mesenchyme transition (EMT) genes including SNAI1, TWIST1, ZEB1, ZO1, MMP1 LAMB3, and LAMC2. Finally, we used RNA-sequencing to identify four additional lncRNAs enriched in UBC patient UEs. These data, suggest that UE-derived lncRNA may potentially serve as biomarkers and therapeutic targets.

## Introduction

lncRNAs, are transcripts that are 5’ 7-methylguanosine capped and either poly-adenylated or unadenylated and are >200 nucleotide long. Once considered genomic noise, lncRNA are proving to be important mediators of normal cellular processes including, developmental imprinting, dosage compensation, and cellular differentiation as well as functions within mature cells such as control of splicing and hormone regulation [1,2]. Dysregulation of lncRNA expression has been shown to be important in malignant processes such as tumor progression [3–6].

With the advent of transcriptome-wide RNA-sequencing (RNA-seq) the discovery of lncRNAs has increased significantly. Recently, Iyer *et al* applied *ab initio* assembly to RNA-sequencing (RNA-seq) libraries from several tumors to reveal thousands of lineage and cancer-associated lncRNAs underscoring the importance of including lncRNA into biomarker and therapeutic target discovery algorithms [7].

One excellent source for biomarker discovery is exosomes. Exosomes are 30-150nM membrane-bound secreted vesicles that are readily purified from culture media and biological fluids including serum, ascites fluid and urine (UEs) [8–14]. Exosomes participate in intercellular communication by delivering proteins, miRNA, mRNA, and lncRNA to recipient cells [15,16].

There is emerging evidence that transcript packaging into exosomes is not stochastic and may rely on signature motifs and secondary structure. [17–20]. Furthermore, oncogenic signaling such as KRAS results in selective packaging of miRNA into exosomes, indicating that cellular transformation may generate a cancer-specific exosome profile that could serve as biomarkers [21].

Notably, quantitative comparisons of producer cells versus exosomes show that exosomes are markedly depleted in mRNA but enriched in lncRNA [18–20]. In addition, lncRNAs show greater specificity than protein-coding mRNA as biomarkers of cancer [22]. Given lncRNA are enriched in exosomes and exhibit cancer specificity they are attractive candidates for biomarker discovery.

Several groups have demonstrated that miRNA, mRNA and protein in serum-derived (SEs) and urinary exosomes (UEs) may serve as biomarkers for both benign and malignant disease [23–36]. For example, we previously identified, epidermal growth factor-like repeats and discoidin I-like domains 3 (EDIL-3) protein in exosomes from urothelial bladder cancer (UBC) cell lines and UEs isolated from UBC patients with high-grade muscle invasive tumors (HGMI pT2-pT4) [34,37].

Prostate and UBC associated lncRNAs have been isolated from voided cells or free floating in urine, and several groups have identified lncRNA in UE from prostate cancer patients [28,38,39]. However, no published studies have demonstrated that UBC UE-derived lncRNAs can serve as biomarkers [28,38,39]. One benefit of using UEs is that the exosome membrane protects the contents from proteases and RNAses, which are ubiquitous in urine [40]. Recent studies have demonstrated that primary UBC tumors contain unique lncRNA, therefore, we sought to capture such lncRNA in UE of UBC patients with HGMI disease [7]. An important member of the class of tumor-associated lncRNAs is HOTAIR, which is overexpressed, by as much as 2000-fold in breast cancer patient tumors compared to normal tissue [3]. HOTAIR over-expression is also associated with increased invasiveness and poor prognosis [3,41]. Importantly, HOTAIR has been shown to regulate several genes involved in epithelial-to-mesenchyme transition (EMT) including Snail family zinc finger 1 (SNAI1), Laminin, beta 3 (LAMB3), Laminin, gamma 2 (LAMC2), Junctional adhesion molecule 2 (JAM2) and ABL proto-oncogene 2 (ABL2) [3,42–44].

The importance of HOTAIR in UBC is starting to come to light through several recent studies. For example, Yan *et al*. demonstrated that elevated expression of HOTAIR predicts high-grade non-muscle invasive UBC (NMIBC) recurrence [45]. They also performed *In vitro* studies to show that HOTAIR is involved in migration and invasion and repression of the canonical Wnt pathway antagonist protein WIF-1 [45].

Martinez-Fernandez *et al*. investigated the possibility that HOTAIR expression could serve as a prognostic marker for disease recurrence in NMIBC [46]. They demonstrated that patients with higher levels of HOTAIR expression also had earlier recurrence of disease. In addition, they showed that Enhancer of Zeste 2 Polycomb Repressive Complex 2 Subunit (EZH2) regulates the expression of HOTAIR. Finally, they used The Cancer Genomic Atlas (TCGA) data set for bladder cancer to show that HOTAIR expression is correlated with stage of UBC with the most invasive T4 tumors having the highest level of HOTAIR expression.

In another study, Sun *et al*. demonstrated that the miR-205 is important for inhibition of proliferation, migration and invasion of UBC cell lines. They identified the cell-cycle regulation gene cyclin J (CCNJ) as a novel target for miR-205. Importantly, they showed that HOTAIR participates in the silencing of miR-205 expression in UBC cells through epigenetic regulation [47].

Taken together these studies demonstrate HOTAIR plays critical roles in UBC. Given the importance of HOTAIR in tumor progression, there is increased interest in using HOTAIR as a biomarker as well as a therapeutic target in cancers in which HOTAIR is aberrantly expressed [2,48,49]. Importantly, having a non-invasive way to detect HOTAIR in cancer patients, such as UEs, would be ideal for biomarker development [15,16,23].

Here, we expand the scope of HOTAIR involvement in UBC biology. For example, we have identified additional EMT factors that are affected by expression of HOTAIR. Critically we show HOTAIR and other lncRNA including tumor-associated lncRNAs HOXA cluster antisense RNA 2 (HOX-AS-2), Antisense non-coding RNA in the *INK4* locus (ANRIL), long intergenic RNA Regulator of Reprogramming (linc-RoR), are overexpressed in UBC cell lines and are enriched in exosomes isolated from UBC cell lines. We also show that HOTAIR, HOX-AS-2, Metastasis-associated lung adenocarcinoma transcript 1 (MALAT1), and lincRoR are overexpressed in tumors and enriched in UEs from UBC patients with HGMI disease (pT2-pT4 on final cystectomy pathology). Importantly, we used RNA-seq to identify additional and novel lncRNAs enriched in UEs from patients with HGMI (pT2-pT4) UBC compared to healthy volunteers. We found four such lncRNAs; Hydatidiform mole associated and imprinted non-protein coding RNA 1 (HYMA1), Long intergenic non-protein coding RNA 477 (LINC00477), LOC 100506688, Orthodenticle homeobox 2 antisense RNA 1 (OTX2-AS1)

## Materials and Methods

### Patients and Volunteers

This study was approved by the Research Subjects Review Board at the University of Rochester Medical Center (RSRB approval number IRB#46706). Chemotherapy naïve patients undergoing cystectomy for HG disease (final cystectomy pathology pT2-pT4) were enrolled in this study. Written informed consent was obtained from all participants, and kept in secure files per RSRB regulations. Research data were coded to ensure that subjects could not be identified, directly or through linked identifiers, in compliance with the Department of Health and Human Services Regulations for the Protection of Human Subjects (45 CFR 46.101(b)). Subject identification numbers were also re-encoded for publication. We collected urine, tumors, and distal normal tissue (DNT) from patients. Tissue was obtained from pathology in formalin fixed paraffin embedded (FFPE) blocks. Tissue was hematoxylin and eosin stained to determine tumor bearing tissue and DNT (at least 3 cm away from the tumor as measured by an independent pathologist). Volunteers were healthy 18+ years old with no history of urologic disease.

### RNA-Sequencing and Data Analysis

RNA quantity was determined using a NanoDrop spectrophotometer and quality was determined with a Agilent Bioanalyzer, using the Pico assay. RNA quality control, library preparation and sequencing were performed by the University of Rochester Genomics Research Center (GRC) using Ribo-depleted cDNA libraries generated by TruSeq RNA kit V2 (Illumina). cDNA was fragmented, barcoded with Illumina-manufactured adaptors, and PCR amplified. Illumina HiSeq2500 was used for high-throughput RNA-Sequencing. Twenty million 125 bp pair-ended reads/sample were obtained.

For NSG data processing; raw 125 bp reads were de-mutiplexed. Low complexity reads and vector contamination were removed. The FASTX toolkit (fast_quality_trimmer) was used to remove bases with quality scores below Q = 13 and aligned to the human genome assembly version hg19/GRCh37 using Burrows-Wheeler Aligner with default settings. Read counts were generated with HTSeq and Cufflinks/Tophat was used for differential expression analysis [50].

### Cell Lines

BC cell lines used: SV-HUC, 5637, TCC-SUP, T24, UMUC3, HT1376, J82, and RT4, obtained from ATCC. HEK293 cells were used for viral packaging. Cells were grown in appropriate media and according to ATCC guidelines. UBC cell line identity was genetically validated by DDC Medical.

### shHOTAIR and shScramble Stable Clones

HEK293 cells were transfected with plasmids: pSPAX2, pMD2G, and GFP-expressing shRNA HOTAIR (shHOTAIR) or Scramble control (shScramble) lentiviral vector constructs, which were generous gifts from Systems BioScience (Mountain View, CA). TCC-SUP and T24 cells were infected with shHOTAIR or shScramble lentivirus and selected by puromycin (2ug/ml) and FACs sorted. Knockdown of HOTAIR was confirmed by qRT-PCR.

### siRNA

T24 cells were plated in 6-well plates at a concentration of 6x10^4^ cells/well, and incubated overnight. Incubation and transfection of siRNA using DharmaFECT 1 reagent (GE Life Sciences, Dharmacon) was performed as per the manufactures protocol. Specifically, for each well, 2.5μL of 5μM siRNA and 1.37μL of DharmaFECT 1 reagent (GE Life Sciences Dharmacon) were used. The siRNA used were previously published: siHOTAIR (siRNA-1 UAACAAGACCAGAGAGCUGUU; siRNA-2 CCACAUGAACGCCCAGAGAUU); and control siGFP (CUACAACAGCCACAACGUCdTdT.) was obtained from GE Life Sciences Dharmacon [51].

### Exosome Preparation and Tracking Analysis

Exosome-producing cell lines TCC-SUP and T24 were grown in Bioflasks as per manufacturer’s recommendations (CELLine 1000AD) in the appropriate culture medium supplemented with 10% exosome-free FBS (EF-FBS). EF-FBS was prepared by ultracentrifugation at 100,000 x g at 4°C for 18 hours.

Exosomes were harvested by serial centrifugation and ultracentrifugation as previously described [34,52]. Exosome protein was quantitated using a microBCA kit (Pierce) and particle analysis was performed using the LM10 nanoparticle characterization system (NanoSight) equipped with the blue laser 488.

### Wound-Healing, Trans-well and 3D Invasion Assays

A wound-healing assay was used to evaluated migration [53]. Wounds were measured at time zero, just after making the wound and fours later. Wound closure was measured using ImageJ software.

A trans-well assay was used to determine invasion as described previously with modifications [54]. 1×10^5^ cells in basal medium were added to 8um pore trans-well inserts (Corning Inc.) pre-coated with reduced growth factor Cultrex (Trevigen Inc). Inserts were harvested, fixed, and stained with 1% toluidine blue, photographed, and the total area of blue-stained cells was calculated using the particle analysis feature of ImageJ software.

For 3D invasion analysis, 1325 cells/190 ul were seeded into 3D mictrotissue ^®^ plates (Sigma) on day 0 [55,56]. After spheroid formation, the media was replaced with basement membrane extract (BME) (Cultrex). Spheroid images were taken using ProgRes^®^ CapturePro 2.7.7 (Jenoptik) viewed with Axio observer A1 microscope (Zeiss). Invasion was measured as length and number of protrusions into the media using ImageJ.

### Western Blotting Analysis

For Western blotting 20ug of exosome or cell lysate protein were analyzed by 10% SDS PAGE. Protein concentration was determined using a microBCA kit (Pierce) as per the manufacturer’s recommendations. Alix (3A9) (Cell Signaling Technology, Cat # 2171, 1:1000 dilution), GAPDH (Santa Cruz Biotechnology, Cat # sc-32233, 1:200 dilution), Snail (C15D3) (Cell Signaling Technology, Cat # 3879, 1:1000 dilution), ZEB1 (H-102) (Santa Cruz Biotechnology, Cat #: Sc-25388, 1:200 dilution), and Beta-Tubulin (Cell Signaling Technology, Cat # 2128, 1:1000 dilution). Anti-Rabbit IgG-horseradish peroxidase, anti-goat IgG-horseradish peroxidase and anti-mouse IgG-horseradish peroxidase were used as secondary antibodies (Santa Cruz Biotechnology). Chemiluminescent detection was performed using Super Signal West Femto Maximum Sensitivity Substrate (Thermo Scientific) as per the manufacturer’s recommendations. Image capture was performed using a ChemiDoc imaging system (Bio-Rad).

### Immunofluorescence

For immunofluorescence of shHOTAIR and shScramble TCC-SUP cells, were fixed in 4% paraformaldehyde and blocked in 0.5% normal goat serum in PBST (0.3% Triton X-100 in PBS) for 1 hour at room temperature. Cells were incubated in ZEB1 (H-102) antibody (Santa Cruz Biotechnology, Cat #: Sc-25388, 1:50 dilution in PBST) overnight at 4°C, washed three times with PBS, then incubated with secondary antibody Goat anti-Rabbit IgG (H+L) Alexa Fluor^®^ 594 conjugate for 2 hours at room temperature (Thermo Scientific Catalog#: A-11012, 1:200 dilution in PBST). After three washes in PBS, stained cells were mounted in VECTASHIELD HardSet Antifade Mounting Medium with DAPI. **ICC imaging:**

Cells were stained and processed simultaneously. Cells were imaged using an FV1000 Olympus laser scanning confocal microscope with a 20x objective in the URSMD Light Microscopy Shared Resource. Laser and voltage settings were adjusted such that the intensity levels of DAPI and Alexa 594 expression were within the linear range for the all images and settings remained identical for all images. Gain and offset were adjusted for the initial control image and thereafter used for all images. An aspect ratio of 1024 x 1024 and a Kalman averaging of 2 were used. All settings were identical for all four groups. The patterns published in this manuscript have been reproduced in two separate experiments and the data represent the reproducible result of treatments and staining for these proteins.

### Electron microscopy

5ul of exosome preparation was placed on 200 mesh copper grids coated with formvar/carbon and incubated 1–2 minutes. Grids were stained with 20ul of 2.0% phosphotungstic acid (pH 6.5) and allowed to dry. A Hitachi 7650 Transmission Electron Microscope at 80kv was used to view samples. Representative electron micrographs were captured using a Gatan Erlangshen 11 megapixel digital camera.

### RNA preparation

Urine was collected from HG patients in the operating room after the induction of general anesthesia. Urine was collected from healthy volunteers at outpatient clinics. Urine was processed immediately for removal of cellular detritus and large extracellular vesicles by serial low-speed and high-speed centrifugation [40]. Urine was then aliquoted into 40ml samples and stored at -80°C until the final pathology was determined.

Only samples from patients who had final cystectomy tumor pathology pT2-pT4 were further processed for RNA using the Urine Exosome RNA Isolation kit as per manufacturer’s instructions (Norgen). RNA was submitted immediately upon isolation for RNA-sequencing. The remaining RNA was stored at -80°C until RNA-sequencing data was made available and RNA was needed for confirmation of RNA-sequencing results.

Cell line exosome RNA was prepared using TRIzol (Thermo Fisher Scientific) as per manufacturer’s recommendations. Cell line RNA was prepared using the Rneasy mini plus kit (Qiagen) as per manufacturer’s recommendations. Tissue RNA was isolated with RNeasy FFPE kit (Qiagen) as per manufacturer’s recommendations. In all cases, RNA was prepared and immediately used for cDNA generation and subsequent qRT-PCR.

cDNA was generated with Bio-Rad iScript cDNA synthesis kit. qRT-PCR was performed with Bio-Rad SYBR-green and Bio-Rad CFX96 Real-Time system. House keeping genes included GAPDH and 18S, both of which are packaged into exosomes [57]. The Normfinder program was used to determine the appropriate housekeeping gene for normalization of qRT-PCR data. Based on evaluation of beta-actin, GAPDH, and 18S, the analysis selected either GAPDH or 18S. The selected housekeeping gene is documented in each figure legend [58]. Primer sequences are listed in S1 Table.

## Results

### UBC cell lines contain tumor-associated mRNA and lncRNA

In order to identify target lncRNA for analysis in UBC we screened selected lncRNAs and mRNAs implicated in tumor progression in other epithelial tumors. We chose five lncRNAs (HOTAIR, HOXA-AS-2, lincRNA—ROR, MALAT1, and ANRIL) and three mRNA Homeobox A13 (HOXA13), SRY-box 2 (SOX2) and POU class 5 homeobox 1 (POU5F1/OCT4) implicated in a range of cancers, including UBC.

For example, HOXA-AS-2 has been demonstrated to repress apoptosis in promyelocytic leukemia cells [59]. The lincRNA—RoR was shown to induce EMT in breast and hepatocellular cancers [60]. Notably HOTAIR was shown to be packaged into exosomes and thereby affect chemosensitivity and survival under hypoxic conditions in recipient cells [16].

MALAT1 has been demonstrated to be a predictor of metastasis in a number of cancers including UBC [61,62]. ANRIL increases cell proliferation and decreases apoptosis in UBC cells [63]. While HOXA13 increases both proliferation and invasion, as well as inhibits apoptosis in glioblastoma multiform cells [64]. The transcription factors SOX2 and OCT4 normally drive the pluripotency of embryonic stem cells but have now been implicated in maintaining cancer stem cells, which may serve as tumor-initiating cells for a large number of tumors [65].

**Fig 1:**
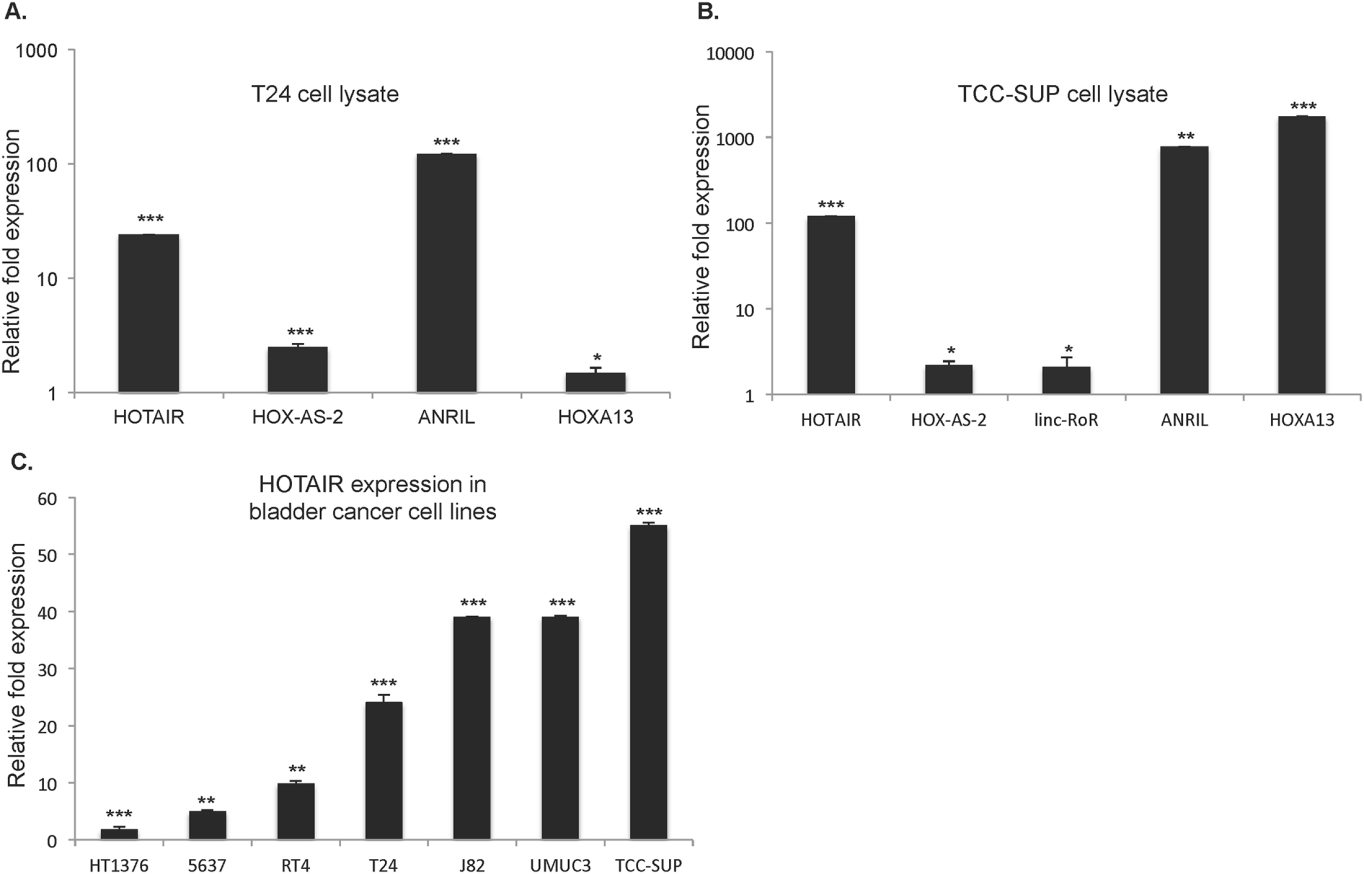
UBC cell lines over-express tumor-associated lncRNA and mRNA relative to the SV-HUC non-tumorigenic urothelial cell line. qRT-PCR was performed on cell lysates from a range of UBC cell lines and the control SV-40 immortalized urothelial cell line SV-HUC. The level of mRNA and lncRNA expression was first normalized to GAPDH and fold-change of gene expression in individual UBC cell lines was compared to the control SV-HUC cells. (A) T24 cell lysates (B) TCC-SUP cell lysates. (C) qRT-PCR of HOTAIR in UBC cell lines normalized to SV-HUC cell line transcript levels. (n = 3 experiments). Student’s t-tests were used for all experiments to identify statistical significance in expression between UBC and control SV-HUC cells (Fig 1A–1C) * p<0.05, ** p<0.001, ***p<0.0001.

In comparison to the level of expression of these selected lncRNA and mRNA in the control SV-40 immortalized non-tumorigenic urothelial cell line SV-HUC, we found elevated expression of HOTAIR, HOXA-AS-2, ANRIL, and HOXA13 in T24 UBC cells. While in TCC-SUP UBC cell line we found HOTAIR, HOX-AS-2, linc-RoR, ANRIL and HOXA13 were elevated (Fig 1A and 1B, respectively).

Given the importance of HOTAIR in tumor progression we evaluated its expression in number of UBC cell lines ranging from Grade II to Grade IV. We found that UBC cell lines have a wide range of HOTAIR expression with TCC-SUP (Grade IV) having the highest and T24 (Grade III) an intermediate level of expression (Fig 1C). These data support recently published observations of the broad range of HOTAIR expression in UBC cell lines [66]. Given that T24 expressed intermediate levels and TCC-SUP expressed relatively higher levels of HOTAIR compared with other UBC cell lines (Fig 1C), we selected these two cell lines to assess the functional roles of HOTAIR in UBC.

### HOTAIR affects UBC cell migration and invasion *in vitro*

**Fig 2:**
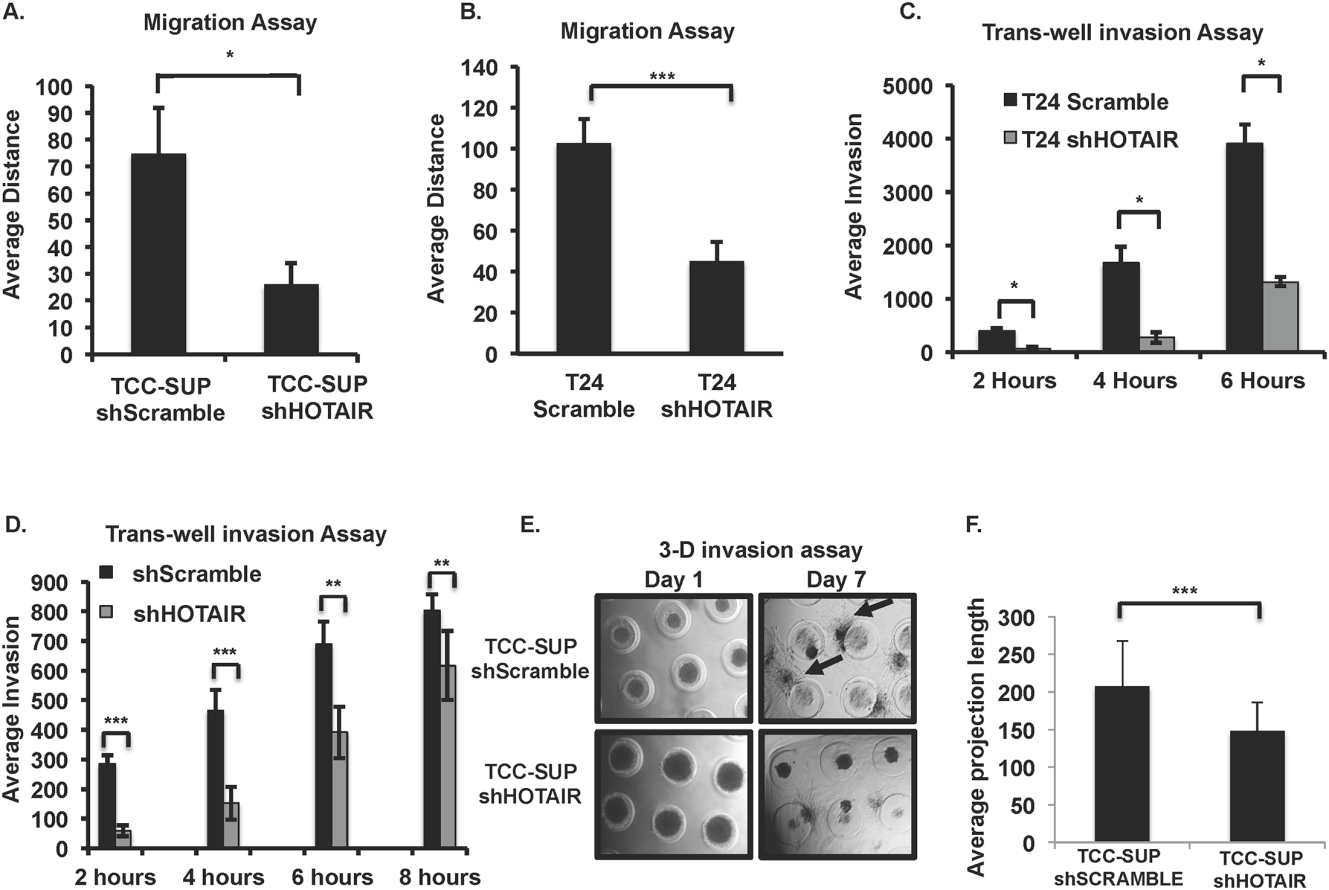
HOTAIR knockdown affects *in vitro* migration and invasion in UBC cells. Distance of migration was measured following scratch-wound assay of lentiviral shRNA knockdown of HOTAIR vs. control shScramble (A) T24 and (B) TCC-SUP UBC cell lines. Trans-well invasion assay of shHOTAIR vs. control shScramble (C) T24 and (D). TCC-SUP cell lines (E) 3-D invasion assay comparing shScramble control TCC-SUP cells to shHOTAIR TCC-SUP cells. Cells are seeded into microtissue ^®^ generated caster gels and allowed to form spheroids. After spheroids are formed, BME is gently layered over the caster gel. Dark circular spheroids are shown and arrows point to projections of invading cells into the surrounding BME. F. Following 7 days of culture, projection lengths were measured from spheroid surface to the distal tip using ImageJ and the average length of projection determined for each cell type. Student’s t-test was used to determine statistical differences in each experiment presented (A-F) *p<0.1, **p<0.05, ***p<0.01 (n = 3–6 experiments/panel).

HOTAIR has been shown to affect migration and invasion in breast, gastric, esophageal and colorectal cancers [3,51,67,68]. In order to demonstrate that HOTAIR has functional roles in UBC, we first generated HOTAIR knockdown cell lines with shRNA against HOTAIR in T24 and TCC-SUP cells. (S1A and S1B Fig, respectively). Knockdown of HOTAIR in T24 and TCC-SUP UBC cell lines led to decreased migration in a standard scratch-wound assay (Fig 2A and 2B, respectively).

**Fig 3:**
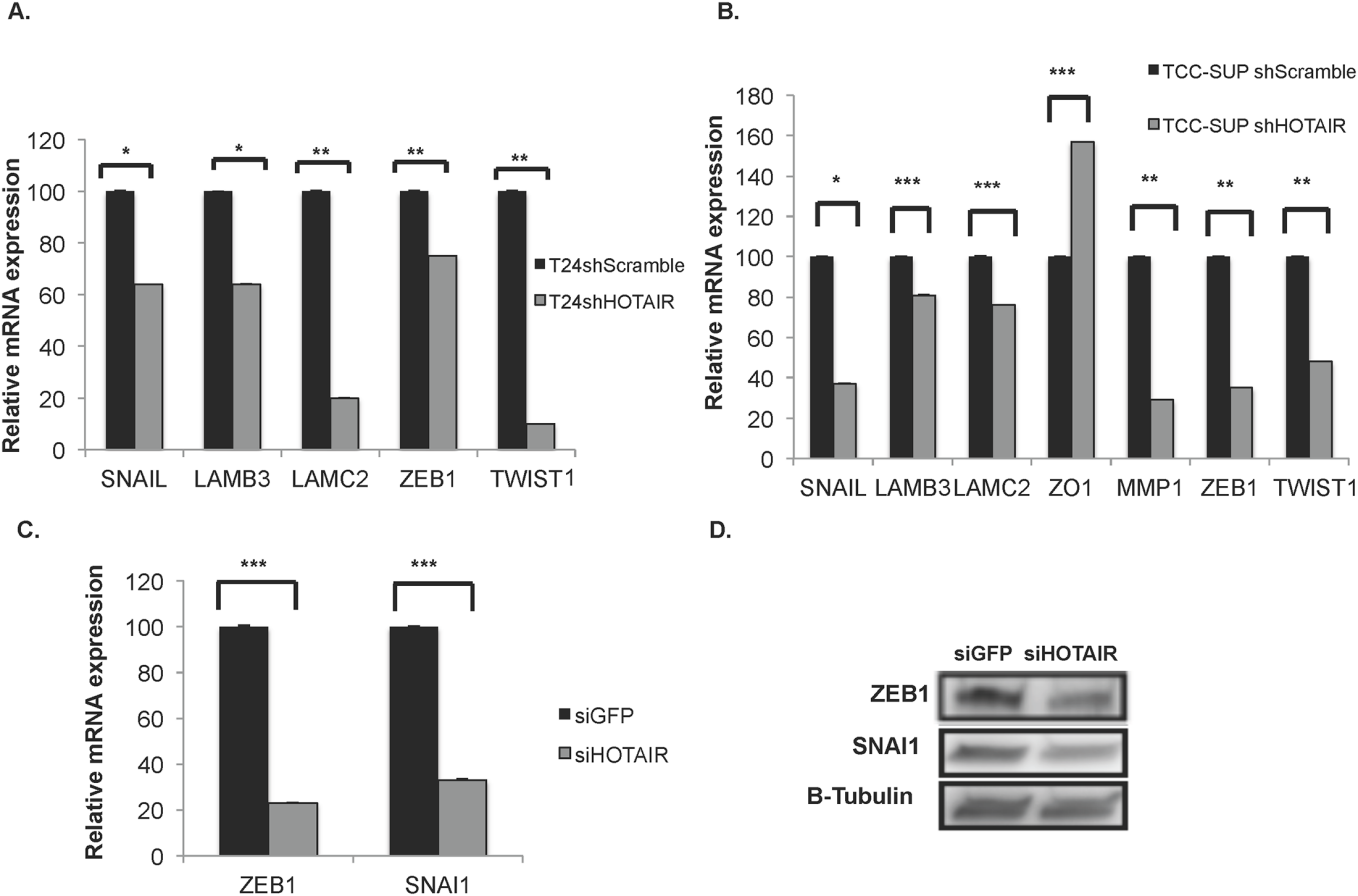
Loss of HOTAIR affects EMT factor expression in UBC cell lines. (A) qRT-PCR of known HOTAIR target genes and classical EMT factors mRNA in shHOTAIR T24 compared to shScramble T24 UBC cells. (B) EMT target gene mRNA expression in shHOTAIR TCC-SUP cells compared to shScramble TCC-SUP cells (in panels A and B EMT mRNA levels were normalized to GAPDH). (C) siRNA targeted against HOTAIR or GFP was used in T24 cells and EMT factors ZEB1 and SNAI1 mRNA expression evaluated by qRT-PCR (mRNA was normalized to 18s). (D) Immunoblot of T24 siGFP and siHOTAIR cells showing reduced protein levels of ZEB1 and SNAI1. GAPDH is a loading control. Beta-actin was used as a loading control. Student’s t-test was used to determine statistical differences between control and HOTAIR knockdown cells in the qRT-PCR experiments presented (A-C) *p<0.1, **p<0.05, ***p<0.01 (n = 3–6 experiments/panel).

Invasion was investigated by both trans-well and 3-D assay systems (microtissues^®^). In 3-D culture tumor cells spontaneously form spheroids. It is well established that cells grown as 3-D spheroids maximize cell-to-cell interactions and mimic more closely endogenous tissue behavior [55,56]. HOTAIR knockdown cells were less invasive in both trans-well (Fig 2C and 2D) and 3-D assays (Fig 2E and 2F).

### HOTAIR affects epithelial-to-mesenchyme transition gene expression

EMT is thought to be an essential oncogenic transition as cancer cells dissociate from epithelial sheets and invade into surrounding tissues. HOTAIR has been shown to affect both activation and repression of numerous EMT pathway-mediating genes in several epithelial cancers [3,69,70]. EMT genes have been demonstrated to regulate tumor invasion in *in vitro* assays such as the trans-well assay and *in vivo* during tumor invasion in every tumor examined [71,72].

We anticipated that the reduced migration and invasion that we observed in the UBC HOTAIR knockdown cells shown in Fig 2A–2F was due to the effects of HOTAIR on EMT gene expression. HOTAIR has been shown to both induce and repress several genes involved in EMT including SNAI1, LAMC2, LAMB3, ABL2, JAM2, PCDHB5, and PCDHB10 [3]. However, HOTAIR does not regulate these genes in every tumor type [48].

To address the HOTAIR/EMT pathway relationship in UBC, we used qRT-PCR to evaluate the HOTAIR target genes previously identified in addition to several other EMT factors in shScramble control cells compared to shHOTAIR UBC cell lines.

Fig 3A and 3B shows that both shHOTAIR T24 and shHOTAIR TCC-SUP cell lines had reduced expression of SNAI1, a master regulator of EMT, as well as EMT pathway genes LAMB3 and LAMBC2 mRNA compared to shScramble controls. However, we did not find that HOTAIR knockdown affected expression of JAM2, ABL2, PCDHB5 or PCDHB 10 (data not shown). Therefore we checked the basal level of expression of these known HOTAIR targets in T24 and TCC-SUP cell (S2 Fig). Although the previously identified targets of HOTAIR were expressed in both T24 and TCC-SUP, their expression was not affected by loss of HOTAIR (data not shown).

Importantly, the expression of two other classical markers of EMT, Zinc-finger E-box binding homeobox 1 (ZEB1), and Twist family BHLH transcription factor 1 (TWIST1) were reduced in the shHOTAIR knockdown UBC cell lines (Fig 3A and 3B).

The expression of E-cadherin (CDH1) and Vimentin (VIM) mRNA was evaluated in shScramble and shHOTAIR T24 and TCC-SUP cell lines. Both CDH1 and VIM mRNA were expressed at extremely low levels and were unchanged between shScramble and shHOTAIR cell lines indicating that HOTAIR most likely does not mediate UBC cell line invasiveness via changes in these two EMT players (data not shown). These data are consistent with previously published reports showing that CDH1 and VIM are minimally expressed in T24 and TCC-SUP [73,74].

Interestingly, Matrix metallopeptidase 1 (MMP1) mRNA expression was reduced in shHOTAIR TCC-SUP UBC cells (Fig 3B). MMP-1 protein cleaves interstitial collagens in the extracellular matrix, thus facilitating tumor invasion [75]. Conversely, we observed increased expression of Tight junction protein 1 (TJP1/ZO1) mRNA in shHOTAIR TCC-SUP UBC cells compared to shScramble control cells (Fig 3B). ZO-1 protein maintains intercellular tight junctions essential for epithelial sheet integrity [76]. The correlation of loss of HOTAIR with increased expression of ZO1 suggests that shHOTAIR TCC-SUP cells are reverting to a more epithelial phenotype. Moreover, these data suggest that HOTAIR may play a role in suppressing some epithelial-related genes in TCC-SUP cells.

To support the results of the shHOTAIR knockdown data we used previously published siRNA directed against HOTAIR to generate siHOTAIR knockdown T24 UBC cells [51] (S3 Fig). Since the expression of SNAI1 and ZEB1 mRNA were both affected by HOTAIR knockdown in T24 and TCC-SUP UBC cells (Fig 3A and 3B, respectively), we evaluated the mRNA (Fig 3C) and protein levels (Fig 3D) of SNA1 and ZEB1 in siGFP and siHOTAIR T24 cells. Both mRNA and protein levels of SNA1 and ZEB1 were reduced in siHOTAIR knockdown T24 cells (Fig 3D). Overall, these data support the correlation between a loss of HOTAIR expression with reduced migration and invasion and EMT factor expression in UBC cell lines.

### HGMI (pT2-pT4) UBC tumors are enriched in tumor-associated lncRNA and mRNA

Tumors and DNT from chemotherapy naïve patients undergoing cystectomy for HG disease (confirmed final pathological stage pT2-pT4) were isolated with IRB approval. Patient clinical characteristics are featured in S2 Table.

**Fig 4:**
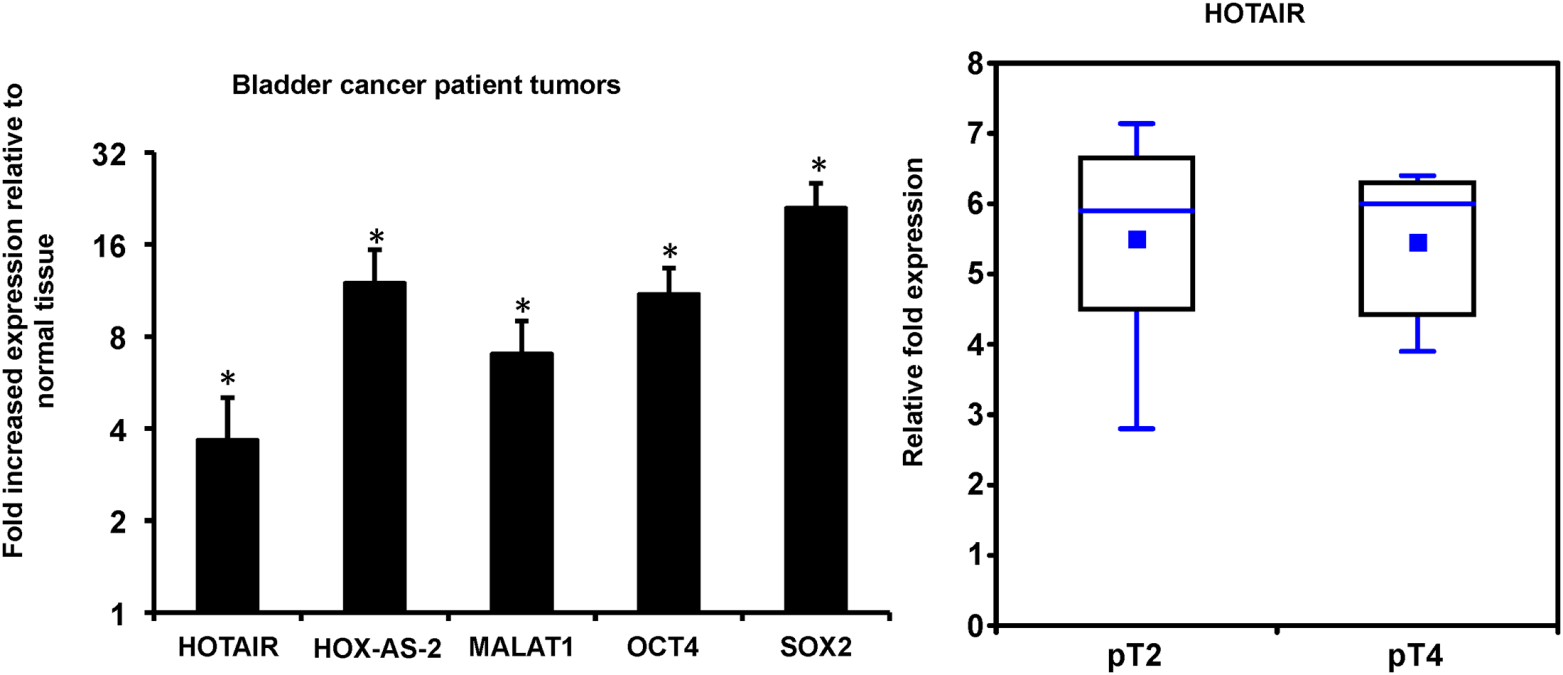
HGMI (pT2-pT4) UBC patient tumors overexpress several tumor-associated lncRNA and mRNA. (A) qRT-PCR of tumor-associated mRNAs and lncRNAs in tumors from patients who underwent cystectomy for HG disease (final pathology pT2-pT4). Tumor and distal normal tissue (DNT) mRNA or lncRNA was normalized to 18s and then the ratio of tumor to DNT was calculated. Statistical significance was determined using Student’s t-test to compare tumor to DNT normalized expression, *p<0.05. (n = 10 patients). (B) HOTAIR was not differentially expressed between pT2 tumors and pT4 tumors (Student’s t-test, p>0.05).

We used qRT-PCR to identify tumor-associated lncRNA and mRNA in tumors compared to DNT epithelium from the same patients (Fig 4A). We found that HOTAIR, HOX-AS-2, MALAT1 and two mRNA OCT4 and SOX2 have higher levels of expression than DNT (the expression level of individual tumors was pooled and compared to pooled DNT expression levels).

Notably, the range of HOTAIR expression overlaps between pT2 and pT4 tumors, this may be due to the small sample size we used in this study (n = 10 patients) (Fig 4B). However, Martinez-Fernandez *et al*. used the TCGA data set for bladder cancer (n = 131 HGMI T2-T4 disease) to show that HOTAIR expression increases with increasing stage of UBC [46]. Nevertheless, our data indicate that HOTAIR expression is increased in HGMI pT2-pT4 tumors relative to DNT.

### UBC cell line exosomes and UEs from patients with HGMI disease (pT2-pT4) contain tumor-associated lncRNAs

Taken together, our work and that of other groups support a role for HOTAIR in UBC. Recent interest in using exosomes for biomarker discovery led us to ask if exosomes produced from UBC cell lines contain lncRNA such as HOTAIR. If so, than these lncRNAs may ultimately be used as biomarkers [42,48,77,78].

**Fig 5:**
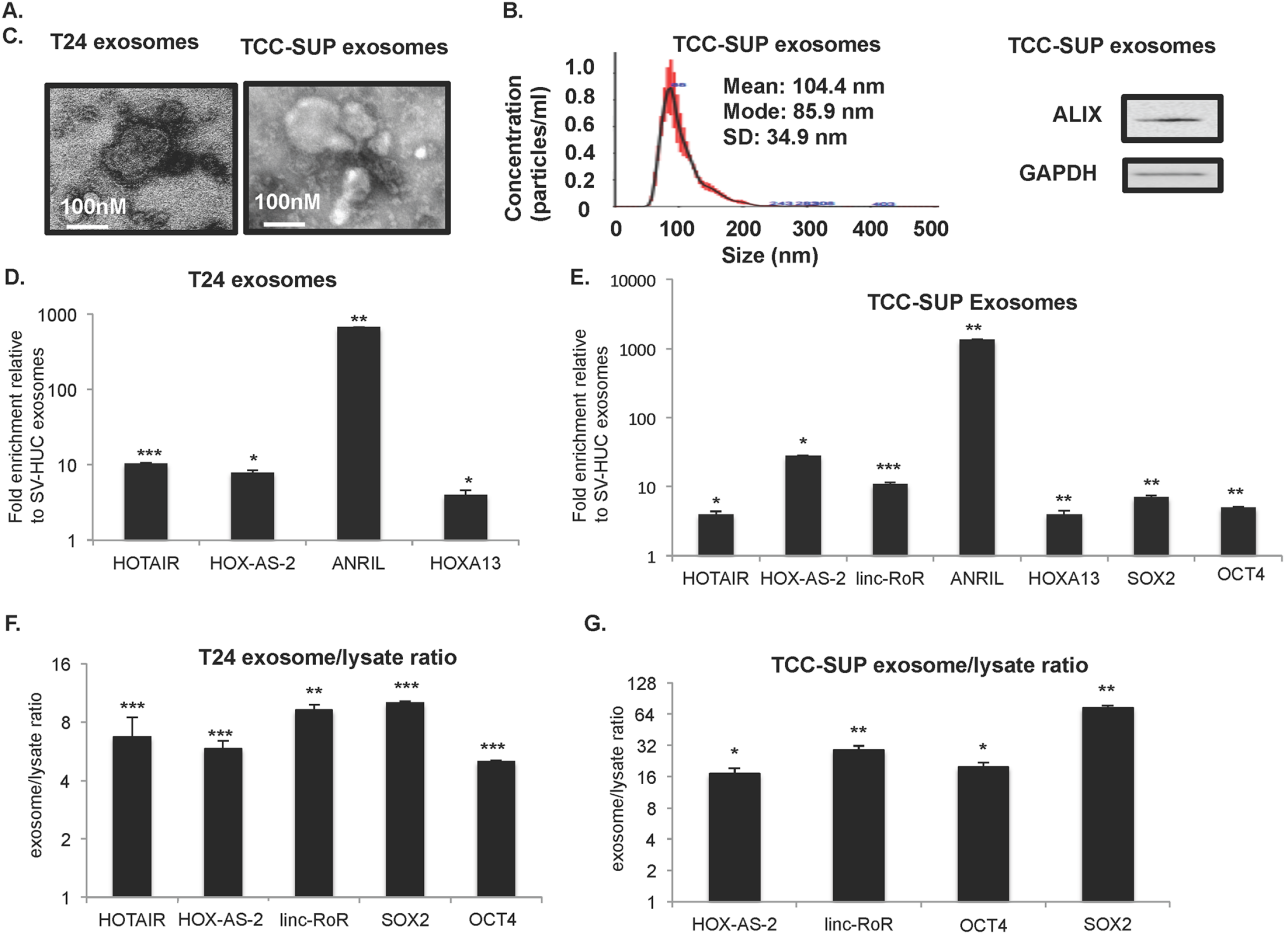
Tumor-associated lncRNAs and mRNAs are enriched in T24 and TCC-SUP exosomes relative to control cell line exosomes. (A) Electron micrograph of exosomes isolated from T24 and TCC-SUP UBC cell lines. White bar 100nm. (B) Particle analysis of TCC-SUP exosomes using the LM10 nanoparticle characterization system (NanoSight). Particles were observed in the size range of 30-150nm (n = 3 experiments). (C) Western blot of exosomes isolated from TCC-SUP UBC cell line. ALIX is a well-characterized exosome marker and GAPDH is a loading control. (D-E) The level of exosomal mRNA and lncRNA expression was normalized to 18S and fold-change of gene expression in T24 and TCC-SUP exosomes was compared to control SV-HUC cell exosomes (D) T24 exosomes and (E) TCC-SUP exosomes. (n = 3 for each experiment). Student’s T-test was used to determine statistical significance between UBC and control exosomes (*p<0.1, **p<0.05, ***p<0.01). (F-G) UBC cell line exosomes are enriched in tumor-associated lncRNAs and mRNAs relative to their producer cell lysates. The ratio of 18S-normalized exosome to cell lysate lncRNAs and mRNAs is shown for (F) T24 and (G) TCC-SUP.

We started with T24 and TCC-SUP cell line exosomes. Fig 5A shows electron micrograph (EM) images of exosomes from T24 and TCC-SUP cell lines and depicts rounded vesicles of ~100 nm, the expected exosome size range. The NanoSight nanoparticle characterization system confirmed a population of particles in the exosome size range of 30–150 nm (Fig 5B). A representative Immunoblot of TCC-SUP exosomes demonstrates the presence of ALIX, a well-established exosome marker (Fig 5C) [34].

Next we used qRT-PCR to identify several tumor-associated mRNAs and lncRNAs enriched in exosomes isolated from T24 and TCC-SUP cells compared to control exosomes from SV-HUC cell line (Fig 5D and 5E, respectively). Importantly, the transcript profile of exosomes overlaps with that of the parental cell lines, suggesting that exosome lncRNA and mRNA content reflect that of their cells of origin and may ultimately serve as biomarkers (compare Figs 1A and 1B to 5D and 5E).

There is growing evidence that transcript packaging into exosomes is not stochastic, therefore, we evaluated whether or not selected lncRNA and mRNA are enriched in exosomes relative to cellular lysate (Fig 5F and 5G). We found that T24 cell line exosomes are enriched in lncRNA HOTAIR, HOX-AS-2 and linc-RoR and mRNA SOX2 and OCT4 compared to T24 cellular lysate transcript levels. While TCC-SUP exosomes contained HOTAIR (Fig 5E), they were not enriched in HOTAIR relative to their cellular lysates. However, we did see an enrichment of linc-RoR, OCT4 and SOX2 in TCC-SUP exosomes relative to TCC-SUP cellular lysate. These data are consistent with previous studies that suggest that lncRNA can be enriched in exosomes relative to lysates [18–20].

**Fig 6:**
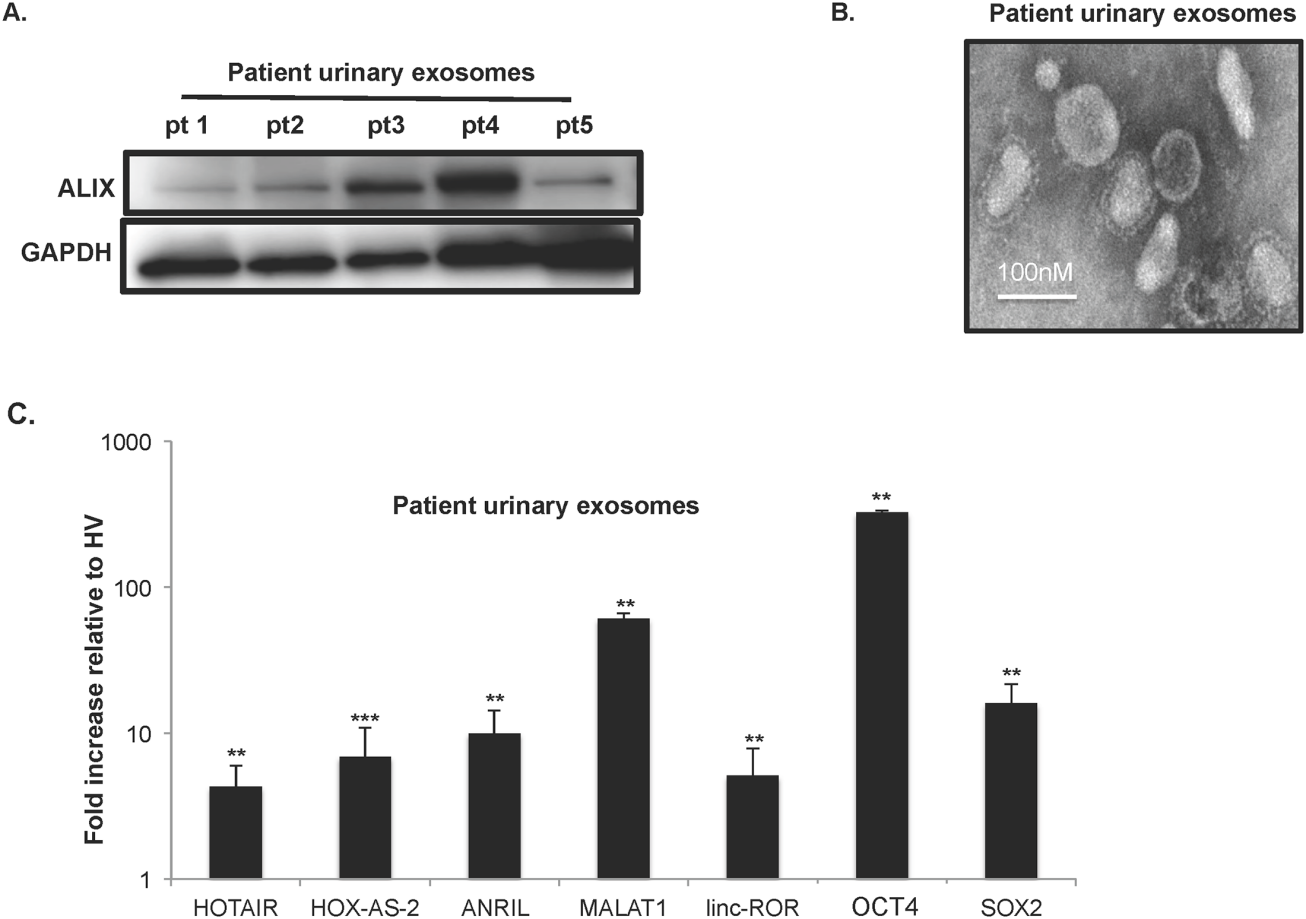
Exosomes isolated from the urine of HGMI (pT2-pT4) UBC patients are enriched in tumor-associated lncRNA and mRNA. (A) Western blot of UEs from five patients. ALIX is a well-known exosome marker, and GAPDH is a loading control. (B) Representative electron micrograph of exosomes purified from the urine of a UBC patient with HGMI disease (pT2-pT4 final pathology). (C) qRT-PCR of UEs for tumor-associated mRNA and lncRNA from UBC patients (n = 8) and HVs (n = 5) and converted to cDNA. qRT-PCR was performs for mRNA and lncRNA. Patient samples were normalized to 18s and HVs normalized to 18s. Student’s t-test **p<0.01 and ***p < .001.

Next we asked if tumor-associated lncRNA and mRNA could be isolated from UEs of UBC patients with HGMI (pT2-pT4) disease. Fig 6A is an immunoblot of UEs from 5 patients. Alix is well-known exosome marker and GAPDH is a loading control (21). Fig 6B is an EM of a UBC patient’s UEs and demonstrates the classic rounded vesicles of the appropriate size range for exosomes ~100nm (white bar).

mRNAs and lncRNAs: HOTAIR, HOX-AS-2, MALAT1, SOX2, OCT4, which were shown to be expressed in UBC patient’s tumors were also enriched in their UEs relative to HVs UEs (Fig 6C). Taken together, these data suggest that the transcript content of UBC patient UEs reflects the transcriptional signature of tumors suggesting that lncRNA in UEs may serve as biomarkers for UBC.

### UBC patient urinary exosomes contain elevated levels of novel lncRNAs

In order to identify additional and novel lncRNA in UEs, we RNA-sequenced UEs from 8 patients with HGMI disease (pT2-pT4 final cystectomy pathology) and 3 HVs. 20 million 125bp pair-ended reads were obtained for each sample.

We identified four lncRNAs (HYMA1, LINC00477, LOC100506688, and OTX2-AS1), two of which are novel (LINC00477, LOC100506688).

**Fig 7:**
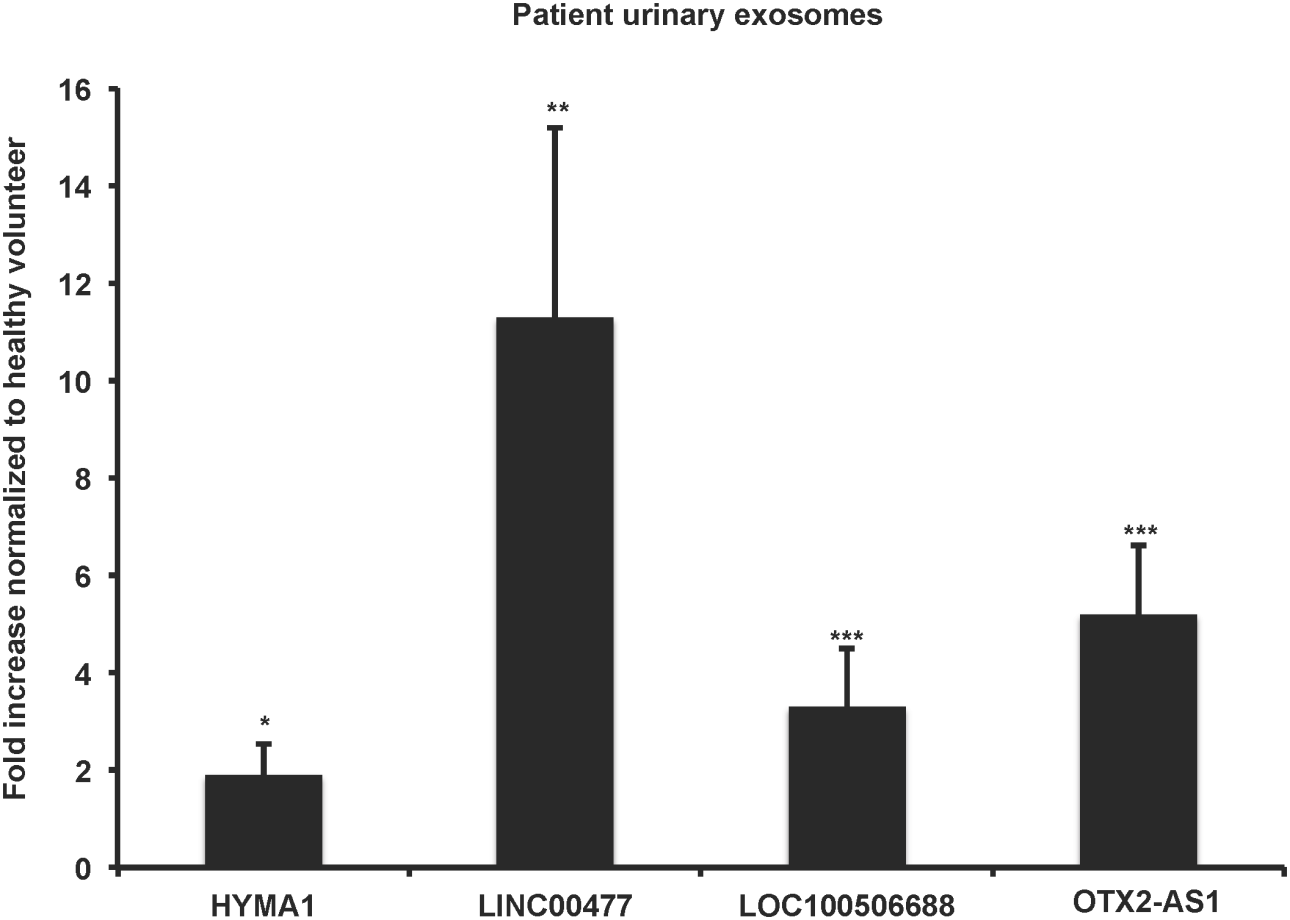
lncRNAs HYMA1, LINC00477, LOC100506688 and OTX2-AS1 are enriched in UEs of UBC patients with HGMI disease (pT2-pT4). Confirmatory qRT-PCR of the novel lncRNAs (normalized to 18s) identified by RNA-sequencing in n = 8 UEs from patients with HGMI (pT2-pT4) UBC and UEs from n = 7 HVs. Student’s t-test *p<0.05, **p<0.01, ***p<0.001.

We used qRT-PCR to confirm the RNA-seq data in the original 8 patients plus two additional patients UEs (n = 10 UBC patients UEs) compared to the original 3 HVs UEs and additional 4 HVs UEs (n = 7 HVs UEs). Fig 7 demonstrates that UEs from patient with HGMI (pT2-pT4) disease are enriched in lncRNA HYMA1, LINC00477, LOC100506688 and OTX2-AS1. These data further support the idea that UEs from UBC patients contain lncRNA and may ultimately serve a role in biomarker discovery.

## Discussion

There is growing interest in the roles that lncRNAs play in tumor initiation and progression. lncRNA have been implicated in tumor progression through their effects on epigenetic modifying complexes, cellular senescence, response to chemotherapeutics, regulation of EMT, response to hypoxia and ability to serve as miRNA sponges [5,44,67–70]. As more lncRNAs are discovered and their biological functions elucidated, lncRNAs will have increasingly important diagnostic and prognostic value as biomarkers [79].

lncRNAs such as HOTAIR are well known for the roles they play in tumor progression making them ideal as therapeutic targets as well as biomarkers. RNA based therapeutic technologies against pathological lncRNA, such as anti-sense oligonucleotides (ASOs), are promising and appealing for intravesical therapy for UBC.

In this study we identify HOTAIR as an important mediator of *in vitro* migration and invasion in UBC cell lines. We show that in addition to the known EMT targets of HOTAIR regulation (SNAI1, LAMB3, LAMC2 [3] ZO1, MMP1 and the classical EMT factors ZEB1 and TWIST1 are also affected by HOTAIR expression (Fig 3A–3D). These data expand the known targets of HOTAIR and underscore the importance of HOTAIR in UBC. Moreover, HOTAIR is overexpressed patient tumors [17,45,47] suggesting that targeting HOTAIR in selected patients may serve therapeutic benefits.

HOTAIR and other lncRNAs have been isolated from SEs and as free-circulating transcripts in patients with various solid tumors suggesting HOTAIR may serve as a biomarker [80,81]. Ideally biomarkers obtained by non-invasive means are desirable from the patient standpoint. UE-derived biomarkers are particularly appealing for several reasons including: 1) non-invasive sample collection; 2) they reflect the transcriptional profile of producer cells; and 3) tumors produce abundant exosomes which enriches tumor transcript biomarkers in UE samples. Here we identified HOTAIR and other tumor-associated lncRNA, in cell line exosomes and UEs from patients with HGMI (pT2-pT4) UBC (Figs 5D and 5E, 6C, and 7), supporting the idea that UEs may contain lncRNA for biomarker discovery.

Most likely, no single lncRNA, mRNA, miRNA will stand alone as a biomarker, but instead panels consisting of a collection of several lncRNA, mRNA, miRNA and proteins will be necessary to fully capture the biomarker profile of any disease.

As a first step in identifying novel UE RNAs for future biomarker validation, we performed RNA-seq on UEs of 8 patients with HGMI (pT2-pT4) disease and UEs from 3 HV controls. RNA-seq provides excellent transcript detection and quantification sensitivity, combined with the ability to identify insertion and deletions, as well as alternatively spliced and polyadenlyated RNA isoforms. Thus RNA-seq of UEs may allow detection of critical cancer-specific transcripts missed by previous microarray studies of UE content [82–84].

Here we identified four additional lncRNAs: HYMAI, LINC00477, LOC100506688 and OTX2-AS1 in UEs from UBC patients (Fig 7). These lncRNAs were confirmed by qRT-PCR in the original 8 patients and 3 HVs plus an additional 2 patients and 4 volunteers. Of course validation in a larger appropriate patient population and comparison with UE isolated from patients with low-grade non-muscle, high-grade non-muscle invasive disease is necessary before these transcripts can be considered biomarkers of HGMI (pT2-pT4) disease. Nevertheless, this work lays the foundation for discovery of lncRNA in UEs from UBC patients.

UEs derive from all organ systems in the body making them ideal for global biomarker discovery [85–92]. One important possibility is that the identification of disease-specific biomarkers in UEs will consist of a mix of tumor-derived and systemic exosomes, therefore, UEs can serve as an unbiased source for biomarker discovery.

## Supporting Information

S1 Fig shRNA lentiviral knockdown efficiency of HOTAIR. Lentiviral shRNA was used to knockdown HOTAIR in T24 UBC cells with an efficiency of 59% (A) and (B) 93% in TCC-SUP UBC cells relative to control scrambled shRNA cells as determined by qRT-PCR (HOTAIR was normalized to 18s). Student’s t-test **p<0.01.
(TIFF)

S2 Fig Known HOTAIR EMT targets are expressed in UBC cell lines. Total RNA was harvested from T24 and TCC-SUP cells and converted to cDNA. qRT-PCR was performed using primers against known EMT targets of HOTAIR regulation [3]. Target transcripts were normalized to GAPDH.
(TIFF)

S3 Fig siRNA HOTAIR knockdown efficiency in T24 UBC cells. The level of HOTAIR was assessed in T24 cells following transient transfection of either control siGFP or two independent siRNAs targeting human HOTAIR. The percent of remaining HOTAIR in siHOTAIR cells is shown (HOTAIR was normalized to 18s).
(TIFF)

S4 Fig Knockdown of HOTAIR reduces ZEB1 immunofluorescence. (A) ZEB1 immunofluorescence in shHOTAIR or shScramble TCC-SUP cells. Left panels are anti-ZEB1, middle panels are DAPI-stained and right panel is the merged image. (B) ZEB1 immunofluorescence in siHOTAIR knockdown or siGFP control T24 cells. Left panels are anti-ZEB1, middle panels are DAPI-stained and right panel is merged. Scale bar is 50μm.
(TIFF)

S1 Table Primer sequences. List of forward and reverse primer sequences used in this study.
(DOCX)

S2 Table Demographic and clinical characteristics of the patient population used in this study. Age, gender, original TURBT tumor pathology is included as well as final cystectomy pathology in chemotherapy naïve patients. Only one patient received adjuvant chemotherapy.
(DOCX)
