# Supplementary figures and images for: Expression of the Long Non-Coding RNA HOTAIR Correlates with Disease Progression in Bladder Cancer and Is Contained in Bladder Cancer Patient Urinary Exosomes
Source: PLoS One. 2016 Jan 22;11(1):e0147236. doi: 10.1371/journal.pone.0147236 (PMC4723257; doi:10.1371/journal.pone.0147236)

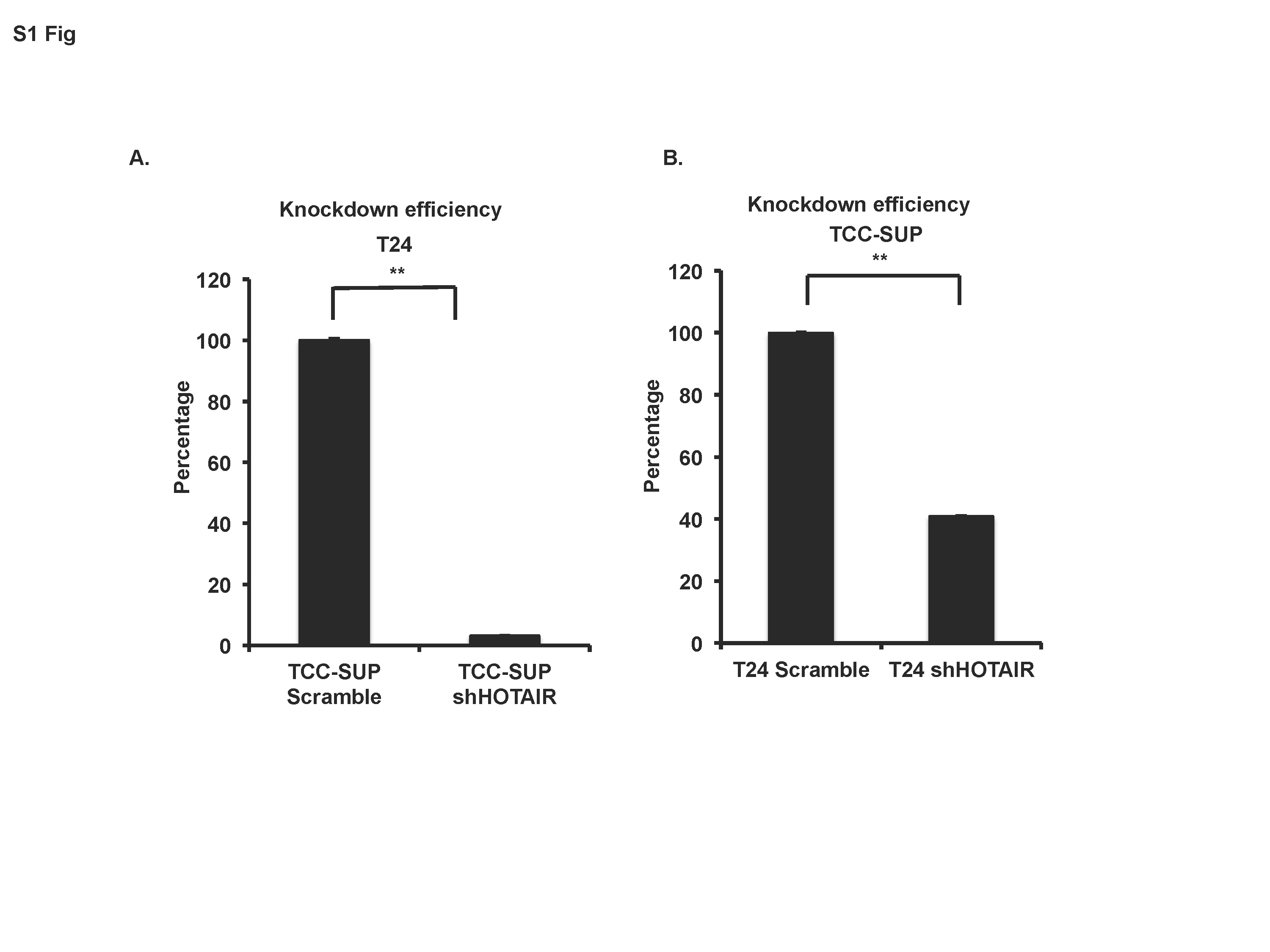

Supplement: S1 Fig — Lentiviral shRNA was used to knockdown HOTAIR in T24 UBC cells with an efficiency of 59% (A) and (B) 93% in TCC-SUP UBC cells relative to control scrambled shRNA cells as determined by qRT-PCR (HOTAIR was normalized to 18s). Student’s t-test **p<0.01. (TIFF) [file pone.0147236.s001.tiff]

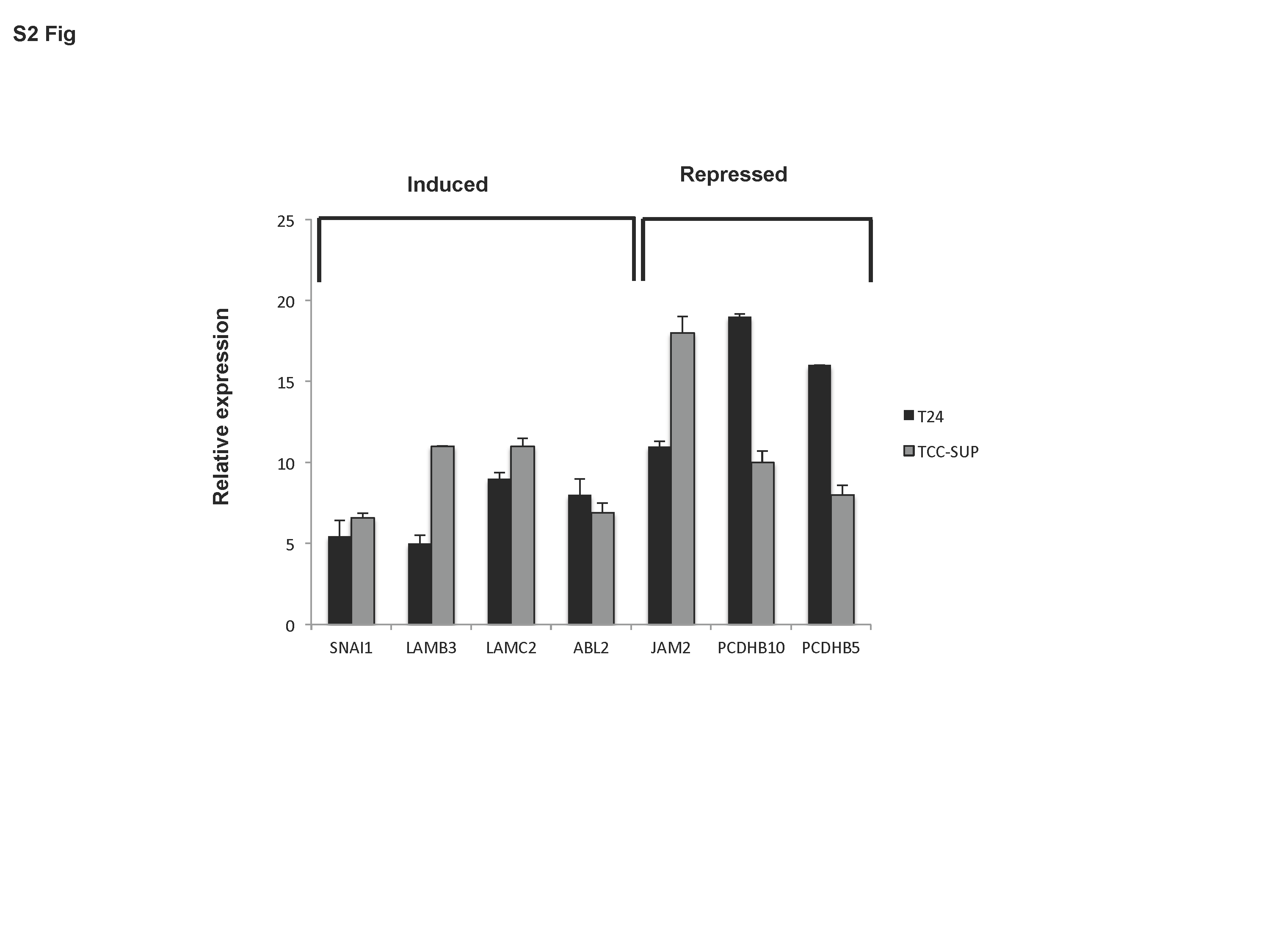

Supplement: S2 Fig — Total RNA was harvested from T24 and TCC-SUP cells and converted to cDNA. qRT-PCR was performed using primers against known EMT targets of HOTAIR regulation [3]. Target transcripts were normalized to GAPDH. (TIFF) [file pone.0147236.s002.tiff]

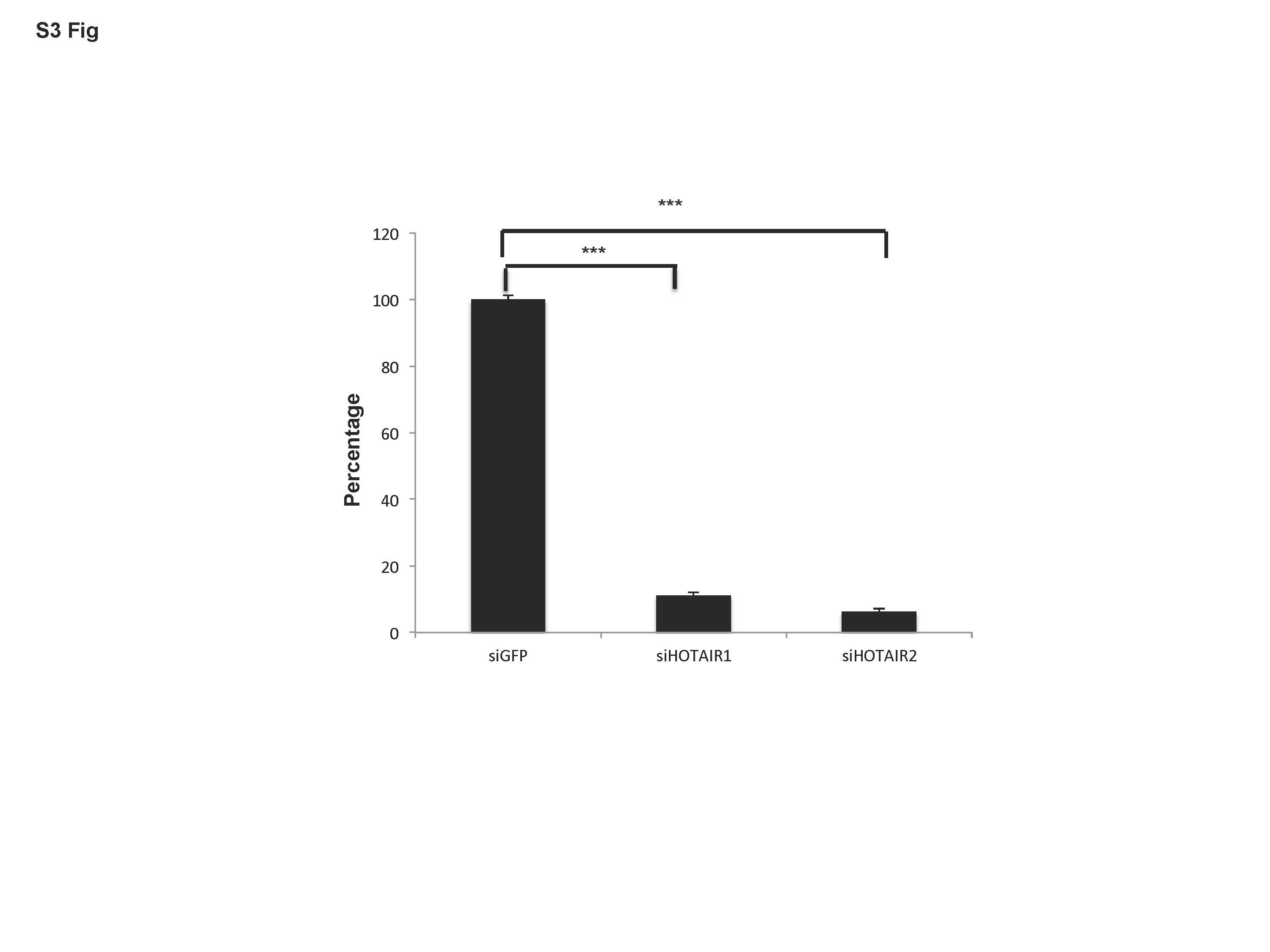

Supplement: S3 Fig — The level of HOTAIR was assessed in T24 cells following transient transfection of either control siGFP or two independent siRNAs targeting human HOTAIR. The percent of remaining HOTAIR in siHOTAIR cells is shown (HOTAIR was normalized to 18s). (TIFF) [file pone.0147236.s003.tiff]

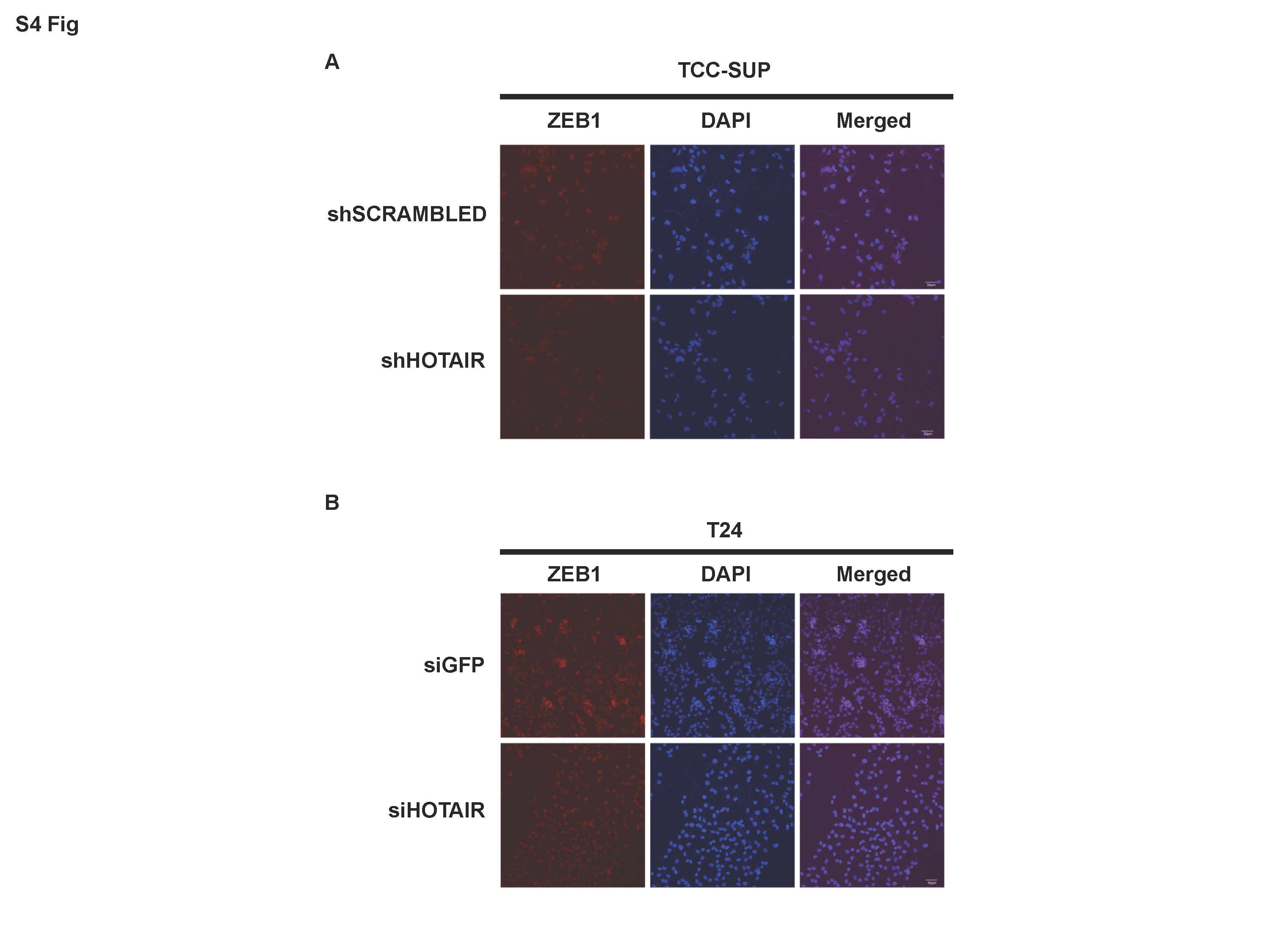

Supplement: S4 Fig — (A) ZEB1 immunofluorescence in shHOTAIR or shScramble TCC-SUP cells. Left panels are anti-ZEB1, middle panels are DAPI-stained and right panel is the merged image. (B) ZEB1 immunofluorescence in siHOTAIR knockdown or siGFP control T24 cells. Left panels are anti-ZEB1, middle panels are DAPI-stained and right panel is merged. Scale bar is 50μm. (TIFF) [file pone.0147236.s004.tiff]
