# Supplementary material for: Expression of the Long Non-Coding RNA HOTAIR Correlates with Disease Progression in Bladder Cancer and Is Contained in Bladder Cancer Patient Urinary Exosomes
Source: PLoS One. 2016 Jan 22;11(1):e0147236. doi: 10.1371/journal.pone.0147236 (PMC4723257; doi:10.1371/journal.pone.0147236)
Supplement: S1 Table — List of forward and reverse primer sequences used in this study. (DOCX) [file pone.0147236.s005.docx]

**Supplementary Figure 1**

| Primer | Forward sequence | Reverse sequence |
| --- | --- | --- |
| SNAI1 | 5’-TGACCTGTCTGCAAATGCTC-3’ | 5’-CAGACCCTGGCTGCTTCAA-3’ |
| LAMB3 | 5’-GCCACATTCTCTACTCGGTGA-3’ | 5’-CCAAGCCTGAGACCTACTGC-3’ |
| LAMBC2 | 5’-CTCTGCTTCTCGCTCCTCC-3’ | 5’-TCTGTGAAGTTCCCGATCAA-3’ |
| ZO1 | 5’-CGGTCCTCTGAGCCTGTAAG-3’ | 5’-GGATCTACATGCGACGACAA-3’ |
| MMP1 | 5’-GAGATCATCGGGACAACTCTCCTT-3’ | 5’-GTTGGTCCACCTTTCATCTTCATCA-3’ |
| HOTAIR | 5’-TCCCCTACTGCAGGCTTCTA-3’ | 5’-CCTAATATCCCGGAGGTGGCT-3’ |
| HOXA-AS2 | 5’-GCTCTCTCCTGCCTTCCTG-3’ | 5’-AGCTTGGCCTACTGTGGAAA-3’ |
| MALAT1 | 5’- GGCCTACTGGGCTGACATTA-3’ | 5’-CTGCCAGGCTGGTTATGACT-3’ |
| OCT4 | 5’-GAAAGCGAACCAGTATCGAGAAC-3’ | 5’-CCCCTGAGAAAGGAGACCCA-3’ |
| SOX2 | 5’-ACCAGCTCGCAGACCTACAT-3’ | 5’-TGGAGTGGGAGGAAGAGGTA-3’ |
| Linc-RoR | 5’CTGGCTTTCTGGTTTGACG-3’ | 5’-CAGGAGGTTACTGGACTTGGAG-3’ |
| ANRIL | 5’-GCCTCATTCTGATTCAACAGC-3’ | 5’-GATCTCCCCGGTTTTCTTCT-3’ |
| HOXA13 | 5’-GCTTCTTTCCCCTCCTA-3’ | 5’-CCAAATGTACTGCCCCAAAG-3’ |
| HYMA1-CE | 5’-TTGCCTTTGTTTTCCTCCAG-3’ | 5’-ACGCAATTGAATGGGAAAG-3’ |
| LINC00477-CE1 | 5’-CTCCTCCTCCTTGGCCTACT-3’ | 5’-CAGCCTGGACAACAGAGTGA-3’ |
| LOC100506688 | 5’-GTGGCTGAGAGGCTACAGGA-3’ | 5’-GAGGTCTGTCGCTGGACTTT-3’ |
| TWIST1 | 5’-TGTCCGCGTCCCACTAGC-3’ | 5’-TGTCCATTTTCTCCTTCTCTGGA-3’ |
| Vimentin | 5’-CGAAAACACCCTGCAATCTT-3’ | 5’-ATTCCACTTTGCGTTCAAGG-3’ |
| ABL2 | 5’-GGACACTTCACTTTGGTGCC-3’ | 5’-TAGTGCCTGGGGTTCAACCT-3’ |
| JAM2 | 5’-TCTTTTGGGGCAGAAAAC-3’ | 5’-AAGATGGCGAGGAGG-3’ |
| ZEB1 | 5’-GCCAATAAGCAAACGATTCTG-3’ | 5’-TTTGGCTGGATCACTTTCAAG-3’ |
| PCDHB10 | 5’-CCCGTCTACACTGTGTCCCT-3’ | 5’-GGAGTACACGACCTCACCGT-3’ |
| PCDHB5 | 5’-AGGTGTGTTTTGACCGGAGAC-3’ | 5’-TCCCTATTTATTCACCAGCG-3’ |
| OTX2-AS1 | 5’-TGAAAGCGATGATGATGTCG-3’ | 5’GCAACAAGAGCCAGGTAAGG-3’ |
| GAPDH | 5’-CTCCTCCACCTTGTAGCGTG-3’ | 5’-CATACCAGGAAATGAGCTTGACAA-3’ |
| 18s | 5’-GGCCCTGTAATTGGAATGATGC-3’ | 5’-CCAAGATCCAACTACGAGCTT-3’ |
