# Supplementary material for: Expression of the Long Non-Coding RNA HOTAIR Correlates with Disease Progression in Bladder Cancer and Is Contained in Bladder Cancer Patient Urinary Exosomes
Source: PLoS One. 2016 Jan 22;11(1):e0147236. doi: 10.1371/journal.pone.0147236 (PMC4723257; doi:10.1371/journal.pone.0147236)
Supplement: S2 Table — Age, gender, original TURBT tumor pathology is included as well as final cystectomy pathology in chemotherapy naïve patients. Only one patient received adjuvant chemotherapy. (DOCX) [file pone.0147236.s006.docx]

| **ID** | **Age** | **Sex** | **TURBT tumor grade** | **TURBT tumor stage** | **Adjuvant chemo** | **Cystectomy tumor grade** | **Cystectomy tumor stage** |
| --- | --- | --- | --- | --- | --- | --- | --- |
| 1 | 58 | Male | High grade | T1 | No | High grade | pT2b N0 |
| 2 | 86 | Male | High trade | T2 | Yes | High grade | pT3a N2 |
| 3 | 80 | Female | High grade | T1 | No | High grade | pT2b N0 |
| 4 | 70 | Female | High grade | T1 | No | High grade | pT2b N0 |
| 5 | 85 | Male | High grade | T2 | No | High grade | pT2b N0 |
| 6 | 78 | Male | High grade | T2b | No | High grade | pT3a N0 |
| 7 | 84 | Male | High grade | T2 | No | High grade | pT4a N0 |
| 8 | 69 | Male | High grade | T1 | No | High grade | pT2b N0 |
| 9 | 72 | Female | High grade | T2 | No | High grade | pT3b N0 |
| 10 | 73 | Male | High grade | T1 | No | High grade | pT2 N0 |

**S2 Table. Demographic and clinical characteristics of the patient population used in this study.**
